# Diagnostic and Therapeutic Challenges in Bronchiectasis With Concurrent Allergic Bronchopulmonary Aspergillosis and Non-tuberculous Mycobacterial Infection: A Case Report

**DOI:** 10.7759/cureus.86114

**Published:** 2025-06-16

**Authors:** Mohammad Abed Alhaleem, Muhammed H Hussain, Ahmed Ahmed, Wafa Ahmed, Luxhman Gunaseelan, Andrew Easow

**Affiliations:** 1 Internal Medicine, Corewell Health Dearborn Hospital, Dearborn, USA; 2 Internal Medicine, Wayne State University, Detroit, USA; 3 Critical Care Medicine, Corewell Health, Dearborn, USA

**Keywords:** allergic bronchopulmonary aspergillosis (abpa), bronchiectasis, diagnostic and therapeutic challenges, immunosuppression, non-tuberculous mycobacterial infection

## Abstract

Bronchiectasis is a chronic respiratory disorder that predisposes patients to recurrent infections and airway inflammation. The coexistence of allergic bronchopulmonary aspergillosis (ABPA) and non-tuberculous mycobacterial (NTM) infection in the setting of bronchiectasis presents a significant diagnostic and therapeutic challenge.

This case report describes a 57-year-old male with a history of asthma and bronchiectasis who developed concurrent ABPA and *Mycobacterium chimaera-intracellulare* infection. Management was complicated by the immunosuppressive requirements of ABPA treatment, which posed a risk for exacerbating the NTM infection. The patient was initiated on itraconazole monotherapy with therapeutic monitoring, but subtherapeutic drug levels and treatment discontinuation due to logistical barriers limited its effectiveness. This case underscores the importance of individualized treatment strategies, close multidisciplinary collaboration, and the need for consistent follow-up in patients with overlapping pulmonary conditions. It highlights the critical balance between immunosuppression and infection control in complex bronchiectasis cases, as well as the systemic challenges that can disrupt optimal care delivery.

## Introduction

Bronchiectasis is a chronic pulmonary disorder characterized by permanent dilation of the bronchi, leading to mucus accumulation, chronic inflammation, and recurrent infections [1]. It can result from a variety of causes, including genetic disorders such as cystic fibrosis, recurrent respiratory infections, immune deficiencies, and allergic conditions [2]. The interplay between bronchiectasis and other pulmonary conditions, including non-tuberculous mycobacterial (NTM) infections and allergic bronchopulmonary aspergillosis (ABPA), can significantly complicate management [3].

Importantly, the coexistence of NTM infection and ABPA poses a unique therapeutic challenge. The anatomical abnormalities in bronchiectasis create a microenvironment conducive to microbial colonization and immune dysregulation, serving as a fertile ground for both *Aspergillus* species and NTM organisms [4,5]. While ABPA requires immunosuppressive therapy, often with corticosteroids, NTM infections necessitate antimicrobial treatment and can worsen under immunosuppression, creating a treatment paradox that requires careful balancing of risks and benefits.

ABPA is a hypersensitivity reaction to *Aspergillus* species colonization in the airways, primarily affecting patients with asthma or cystic fibrosis. It is characterized by elevated total and *Aspergillus*-specific IgE levels, eosinophilia, bronchiectasis, and recurrent pulmonary exacerbations [6]. Corticosteroids and antifungal therapy, typically itraconazole, are the cornerstone of ABPA management. However, the coexistence of NTM infection complicates treatment, as corticosteroids can exacerbate mycobacterial infections by suppressing immune function [7].

Pulmonary NTM infections, including *Mycobacterium avium* complex (MAC), are opportunistic infections that frequently occur in patients with underlying structural lung disease, such as bronchiectasis [8]. Diagnosis requires a combination of radiographic findings, microbiological evidence, and clinical symptoms, making it challenging to differentiate from other chronic lung infections. Treatment typically includes a prolonged multidrug regimen, which carries a significant burden in terms of medication side effects and adherence [8].

This case report highlights the diagnostic and therapeutic challenges in a 57-year-old male with bronchiectasis, suspected ABPA, and an initial positive sputum culture for MAC. The patient's complex clinical course underscores the importance of careful treatment selection and close monitoring in the presence of overlapping pulmonary conditions.

## Case presentation

**Figure 1:**
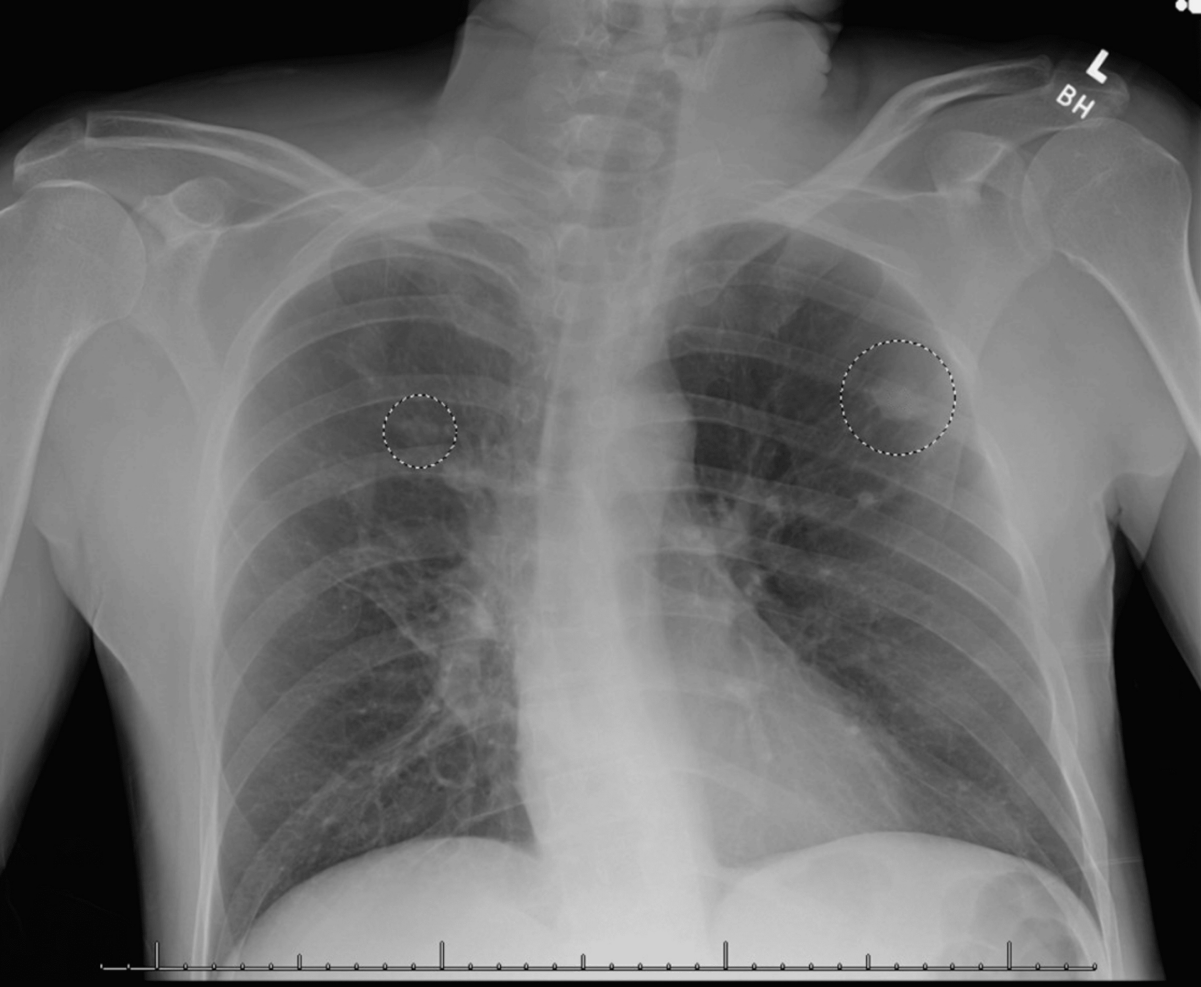
Chest X-ray Note the bilateral pulmonary nodules (circles) measuring 2.5 cm in the right upper lobe and 1.5 cm in the left upper lobe.

A 57-year-old male with a past medical history of asthma and bronchiectasis presented for evaluation following hospitalization due to a traumatic fall from a height of six feet. He sustained multiple injuries, including a right distal radius fracture, right pneumothorax, transverse process fractures, and an acetabular fracture. During his hospitalization, a chest X-ray revealed bilateral pulmonary nodules measuring 2.5 cm in the right upper lobe and 1.5 cm in the left upper lobe (Figure 1).

**Figure 2:**
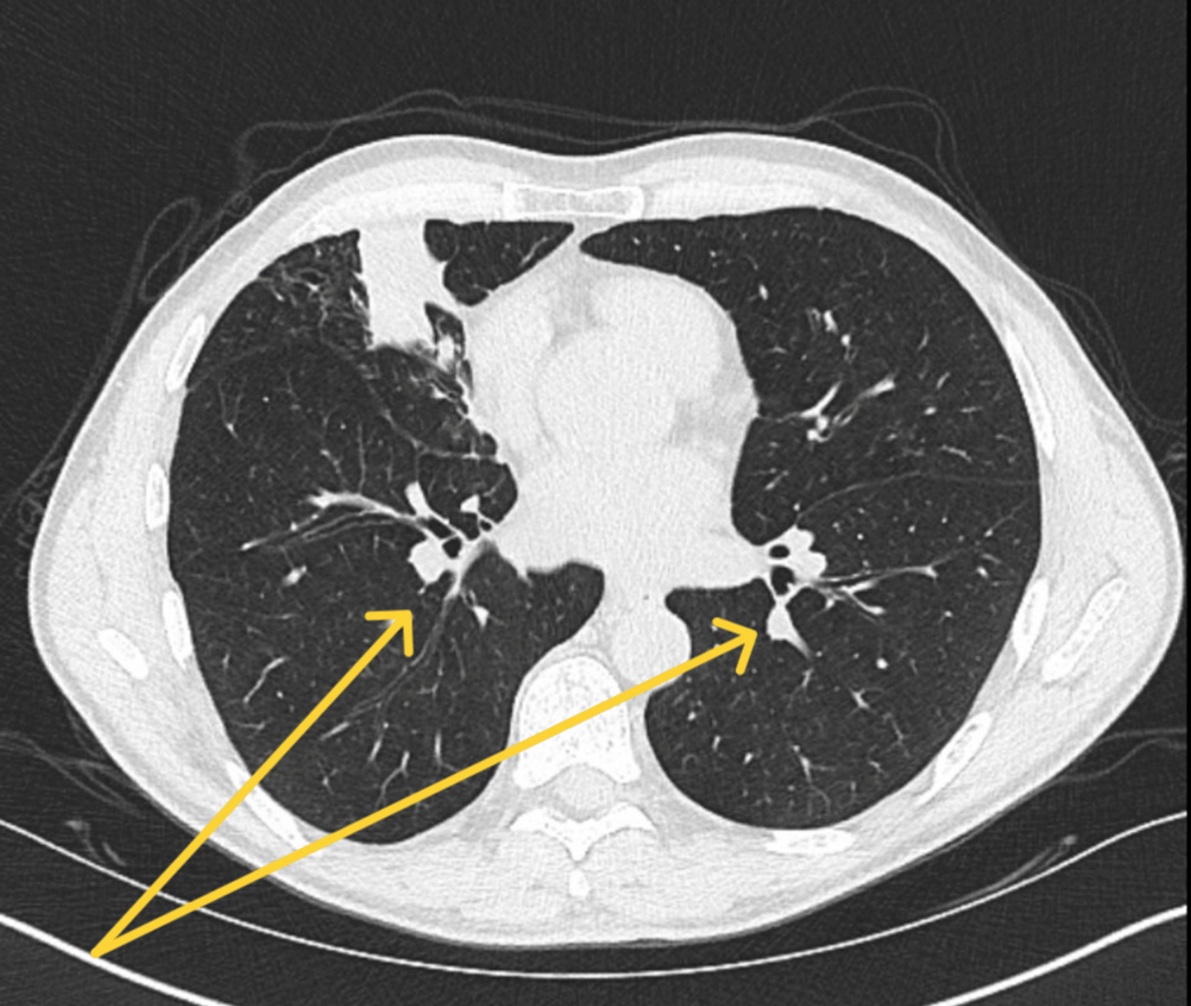
CT Chest With Contrast Yellow arrows: low-density airspace opacities present within the left upper lobe and right middle lobe, with a branching-like appearance.

A CT chest with contrast, performed two years prior, also revealed low-density airspace opacities within the left upper lobe and right middle lobe, which had a branching-like appearance (Figure 2).

The patient was a lifelong non-smoker with no significant occupational exposures. He had a childhood history of asthma but had been asymptomatic in recent years, requiring only occasional use of a rescue inhaler. He denied any history of tuberculosis exposure, illicit drug use, or prior incarceration. He recalled a brief episode of hemoptysis several years prior, along with transient night sweats. His respiratory symptoms were minimal upon admission but evolved to include a mild, productive cough during hospitalization.

An infectious disease consultation raised suspicion for an acute exacerbation of bronchiectasis, prompting initiation of a five-day course of ceftriaxone and doxycycline, with sputum cultures obtained for further evaluation. Given the chronic nature of his imaging findings, the differential diagnoses included a prior untreated infectious process, granulomatous lung disease, and possible NTM infection. Initial workup included AFB sputum cultures, fungal cultures, and serologic studies for tuberculosis and endemic mycoses.

A subsequent pulmonology evaluation confirmed longstanding bronchiectasis with pulmonary nodules, but no prior biopsy or intervention. Additional testing included repeat AFB sputum cultures and fungal serologies. The patient’s asthma remained stable, and there was no evidence of significant worsening of his bronchiectasis at that time. Pulmonology strongly considered initiating corticosteroid therapy for ABPA but was unable to proceed due to the presence of a positive AFB sputum culture, which raised concerns about concurrent NTM infection.

**Figure 3:**
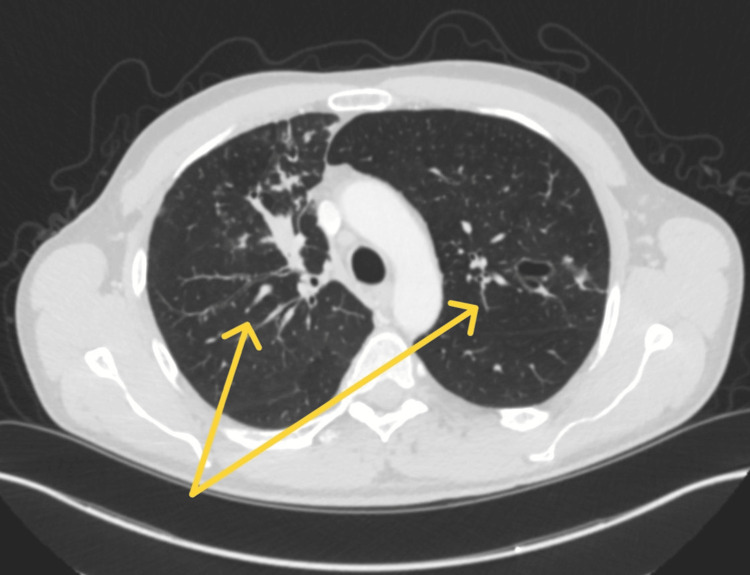
Post-discharge CT Chest With Contrast Yellow arrows: bronchiectasis with associated nodular branching and tree-in-bud opacities, as well as lobular soft tissue nodules in the left upper lobe.

Following hospital discharge, sputum cultures confirmed the presence of the *Mycobacterium chimaera-intracellulare* group, though additional AFB cultures were negative. Laboratory testing also revealed evidence of ABPA, with markedly elevated total IgE and *Aspergillus fumigatus*-specific IgE levels (Table 1). A repeat CT chest with contrast revealed findings of bronchiectasis with associated nodular branching and tree-in-bud opacities, as well as lobular soft tissue nodules in the left upper lobe (Figure 3).

**Table 1:** Laboratory Abnormalities

Test	Value	Reference Range
*Aspergillus fumigatus* specific IgE level	46.3 kU/L	Less than 0.10 kU/L: Class 0, No significant level detected
		0.10-0.34 kU/L: Class 0/1, Clinical relevance undetermined
		0.35-0.70 kU/L: Class 1, Low
		0.71-3.50 kU/L: Class 2, Moderate
		3.51-17.50 kU/L: Class 3, High
		17.51-50.00 kU/L: Class 4, Very High
		50.01-100.00 kU/L: Class 5, Very High
		Greater than 100.00 kU/L: Class 6, Very High
Total IgE level	1827 IU/mL	Less than 165 IU/mL

Given concerns about exacerbating potential NTM infection with corticosteroids, treatment was initiated with itraconazole monotherapy with plans for serial monitoring of IgE levels, itraconazole serum levels, and monthly laboratory assessments.

At subsequent follow-up, the patient reported symptomatic improvement, including reduced cough, improvement in shortness of breath, and weight gain, though he was still not anywhere near what he used to weigh prior to his accident. However, therapeutic monitoring revealed subtherapeutic itraconazole levels, raising concerns about drug absorption and adherence. Despite this, his clinical status remained stable, and additional AFB cultures were negative. However, given his persistent imaging findings and laboratory abnormalities, he still required ongoing treatment for ABPA. Unfortunately, the patient later encountered difficulties obtaining itraconazole due to insurance and logistical challenges, leading to an unintended discontinuation of therapy after completing only 13 to 14 weeks of treatment. He was subsequently lost to follow-up, raising concerns about potential disease progression in the absence of ongoing treatment and surveillance.

**Figure 4:**
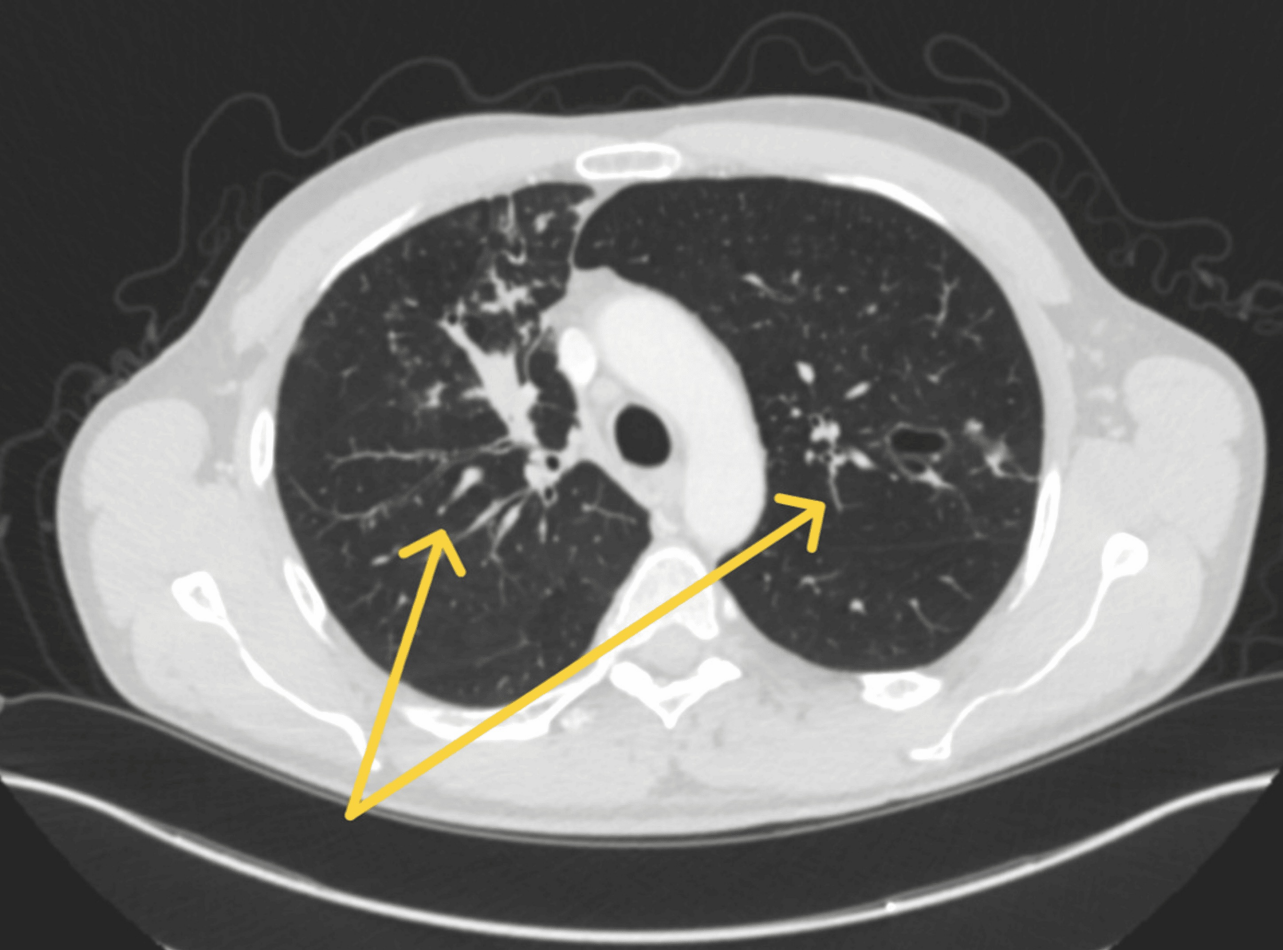
Follow-Up CT Chest Yellow arrows: mild improvement in the overall density of the chronic fungal infection in the right upper lobe. Multiple nodular opacities in the left upper and lower lobes remained stable.

A follow-up CT chest demonstrated only mild improvement in the overall density of the chronic fungal infection in the right upper lobe (Figure 4). Multiple nodular opacities in the left upper and lower lobes remained stable, without new acute findings. Given the limited improvement on imaging and continued laboratory abnormalities, the patient still required ongoing treatment for ABPA.

## Discussion

The coexistence of ABPA and NTM infection in a patient with bronchiectasis represents a particularly challenging clinical scenario. Bronchiectasis provides a structurally abnormal airway that supports persistent microbial colonization, chronic inflammation, and impaired mucociliary clearance. This setting not only predisposes patients to recurrent infections but also acts as a permissive niche for opportunistic pathogens like *A. fumigatus* and MAC [4,5]. The simultaneous presence of these two pathogens introduces therapeutic complexity: corticosteroids, essential for managing ABPA, may exacerbate NTM infections, while delaying immunosuppressive therapy risks worsening ABPA-related inflammation [5]. ABPA is a progressive disease that requires close monitoring and early intervention to prevent irreversible lung damage [6]. The mainstay of treatment is corticosteroids, which effectively reduce airway inflammation and prevent exacerbations. However, their use is contraindicated in the presence of NTM infections due to the risk of promoting mycobacterial proliferation [7]. This limitation necessitates an alternative treatment approach, such as antifungal therapy with itraconazole, which was initiated in this case [3].

Itraconazole has variable absorption and requires therapeutic drug monitoring to ensure efficacy [6]. In this case, the patient had subtherapeutic drug levels despite reported adherence, raising concerns about bioavailability and absorption issues. Factors that can limit the bioavailability of itraconazole include its dependence on gastric acidity for dissolution, interactions with proton pump inhibitors (PPIs) or H2-receptor antagonists that reduce stomach acid, and variable gastrointestinal motility [9]. Additionally, food intake plays a significant role in itraconazole absorption, as the capsule formulation requires an acidic environment and is better absorbed when taken with a meal, whereas the solution formulation is better absorbed in a fasting state [10]. Furthermore, drug-drug interactions with cytochrome P450 3A4 (CYP3A4) inducers, such as rifampin and certain anticonvulsants, can significantly reduce itraconazole levels [11]. In this patient, potential contributing factors, such as concurrent medications, dietary habits, and gastrointestinal health, should be considered when assessing itraconazole absorption. While he was not taking any medications that could reduce the bioavailability and absorption of itraconazole, his premature discontinuation of itraconazole due to insurance difficulties left him at risk for disease recurrence. The mild improvement on CT imaging suggests that, while the therapy had some effect, it was not sustained long enough for complete resolution [2].

Given the persistent bronchiectasis and nodular opacities, a more aggressive treatment approach, including systemic corticosteroids, would typically be considered for ABPA [6]. However, the presence of a prior positive AFB culture led to clinical hesitation in starting steroids. In such cases, a thorough risk-benefit assessment is required, weighing the potential for NTM progression against the consequences of untreated ABPA [8].

Long-term management of ABPA in bronchiectasis patients is critical to prevent further structural lung damage, recurrent exacerbations, and pulmonary decline. This case highlights the importance of regular imaging, laboratory monitoring, and adherence to therapy [8]. Additionally, addressing barriers to medication access is crucial, as treatment interruptions can lead to disease progression and worsened outcomes [2].

This case also reinforces the need for a multidisciplinary approach. Collaboration between infectious disease specialists, pulmonologists, and pharmacists is essential for optimizing antifungal therapy, monitoring drug interactions, and ensuring appropriate therapeutic drug levels [7]. Future studies may explore alternative antifungal agents or steroid-sparing approaches for managing ABPA in the setting of suspected NTM.

## Conclusions

This case underscores the diagnostic and therapeutic complexities involved in managing patients with bronchiectasis, complicated by both ABPA and NTM infection. The overlapping presence of these infections in the structurally compromised airways of bronchiectatic lungs highlights the need for nuanced clinical decision-making. Treatment must delicately balance immunosuppression against infection control. Multidisciplinary care, adherence to therapy, and long-term monitoring are vital to improving outcomes in this vulnerable population.

Future management, if re-engaged, would require reassessment of ABPA activity, the potential need for corticosteroid therapy, and continued monitoring for NTM infection. This case underscores the critical need for patient adherence, timely medication access, and close multidisciplinary collaboration to ensure optimal outcomes in patients with overlapping pulmonary conditions.
